# Dr. PIAS 2.0: an update of a database of predicted druggable protein–protein interactions

**DOI:** 10.1093/database/bas034

**Published:** 2012-10-05

**Authors:** Nobuyoshi Sugaya, Satoru Kanai, Toshio Furuya

**Affiliations:** Drug Discovery Department, Research & Development Division, PharmaDesign, Inc., Hatchobori 2-19-8, Chuo-ku, Tokyo 104-0032, Japan

## Abstract

Druggable Protein–protein Interaction Assessment System (Dr. PIAS) is a database of druggable protein–protein interactions (PPIs) predicted by our support vector machine (SVM)-based method. Since the first publication of this database, Dr. PIAS has been updated to version 2.0. PPI data have been increased considerably, from 71 500 to 83 324 entries. As the new positive instances in our method, 4 PPIs and 10 tertiary structures have been added. This addition increases the prediction accuracy of our SVM classifier in comparison with the previous classifier, despite the number of added PPIs and structures is small. We have introduced the novel concept of ‘similar positives’ of druggable PPIs, which will help researchers discover small compounds that can inhibit predicted druggable PPIs. Dr. PIAS will aid the effective search for druggable PPIs from a mine of interactome data being rapidly accumulated. Dr. PIAS 2.0 is available at http://www.drpias.net.

**Database URL**: http://www.drpias.net.

## Introduction

Modulating protein–protein interactions (PPIs) using small compounds can greatly contribute to the therapeutic intervention of various human diseases, since most proteins in a cell function by interacting with other proteins (1–4). Many proteins such as membrane receptors and enzymes have been intensively studied as drug targets, and various biological information on the target proteins have been stored in some public databases, for example Therapeutic Target Database (5) and SuperTarget (6). In contrast, there are only a limited number of drug target PPIs deposited in databases, TIMBAL (7) and 2P2IDB (8), despite the biological importance of the PPIs.

To date, we have developed novel methodologies to assess the druggability [also called ‘ligandability’ (9)] of PPIs. Our approach is based on support vector machine (SVM) utilizing the physicochemical properties of the PPI-inhibitor-binding pockets (structural attributes), the number of drugs/chemicals that target interacting proteins (drug/chemical attributes) and available information on biological function such as diseases, pathways, gene ontologies and gene expression profiles (functional attributes) (10, 11). By applying our methodologies to human PPIs, we have predicted their druggabilities (11). We created a database of predicted druggable PPIs, named Dr. PIAS, aiming at helping researchers effectively explore druggable PPIs from interactome data (12). Here, we introduce Dr. PIAS version 2.0, which contains novel features not present in the first version of the database.

## Novel Features of DR. PIAS 2.0

### PPI data

We have retrieved PPI data from three resources, mainly focusing on human, mouse, rat and human immunodeficiency virus proteins. One of the resources is the Entrez Gene (13). It integrates PPI data from BIND (14), BioGRID (15) and HPRD (16) databases, and thus includes most human PPIs experimentally identified to date. Other two resources are the Genome Network Platform in Japan (http://genomenetwork.nig.ac.jp/index_e.html) and several publications (17–19), and many PPIs in these resources have not been registered in the Entrez Gene yet. In the current version, Dr. PIAS contains 83 324 PPIs, a considerable increase from the 71 500 PPIs in the previous version. PPIs between human proteins are most abundant (72 130), followed by mouse PPIs (4819).

### New positive instances in our SVM-based method

Dr. PIAS assesses the druggability of PPIs based on one of the supervised machine learning method, SVM, using the computational program package Libsvm (http://www.csie.ntu.edu.tw/∼cjlin/libsvm/). In our previous studies, we used 30 well-studied drug target PPIs as the positive instances in our SVM-based method (11). Progress in the research area of drug target PPIs has allowed us to add 4 PPIs (CREBBP/TP53, MDM4/TP53, RAF1/YWHAZ and S100B/TP53) and 10 tertiary structures (PDB entries 2D82, 2OPY, 2W3L, 3HCM, 3INQ, 3JZK, 3LBJ, 3LBK, 3LBL and 3RDH) as the new positives. These structures are complexes of a protein from a PPI and a compound inhibiting the PPI. Although the number of added PPIs and structures is small, in the cross-validation tests, these additions increase the accuracy and specificity from 80.5% and 80.5% in the previous SVM classifier to 83.2% and 84.7% in the new classifier, respectively. Sensitivity remains constant at 81.6%. Therefore, the new classifier used in Dr. PIAS 2.0 has more discriminative power for druggable and non-druggable PPIs than the previous one.

Application of the new SVM classifier to the positives shows that the mean value of druggability scores (using all attributes) of the positives is 0.908 (Supplementary Table S1). Among 72 096 human PPIs (except for the positives) in Dr. PIAS, 41 have a score ≥0.908 and can thus be considered to be ‘highly druggable’ (Supplementary Table S2).

### Similar positives of druggable PPIs

In the current version, we introduced a novel concept of ‘similar positives’ of druggable PPIs. When a PPI is judged to be highly druggable by our method, the information on which positive is most similar to the PPI will be valuable for further investigation of the PPI from a viewpoint of discovering small inhibitory compounds. As described in our previous papers, we calculated the ‘druggability score’ of a PPI as the number of times the PPI was judged to be positive in a 10 000 times training-prediction iteration using 10 000 random training data (11, 12). A high druggability score of a PPI indicates that the PPI has a feature vector highly similar to the positives. If a PPI (test instance) was judged as positive in a process, we calculated similarities between the feature vector of the test and that of each positive. Similarities were measured using a radial basis function kernel, *k*(**x**_positive_, **x**_test_) = exp (−γ||**x**_positive_ − **x**_test_||^2^) (where γ > 0). When a positive gives the largest *k*(**x**_positive_, **x**_test_) among all measurements, it is identified as most closely located (most similar) to the test in a feature space of SVM.

**Figure 1:**
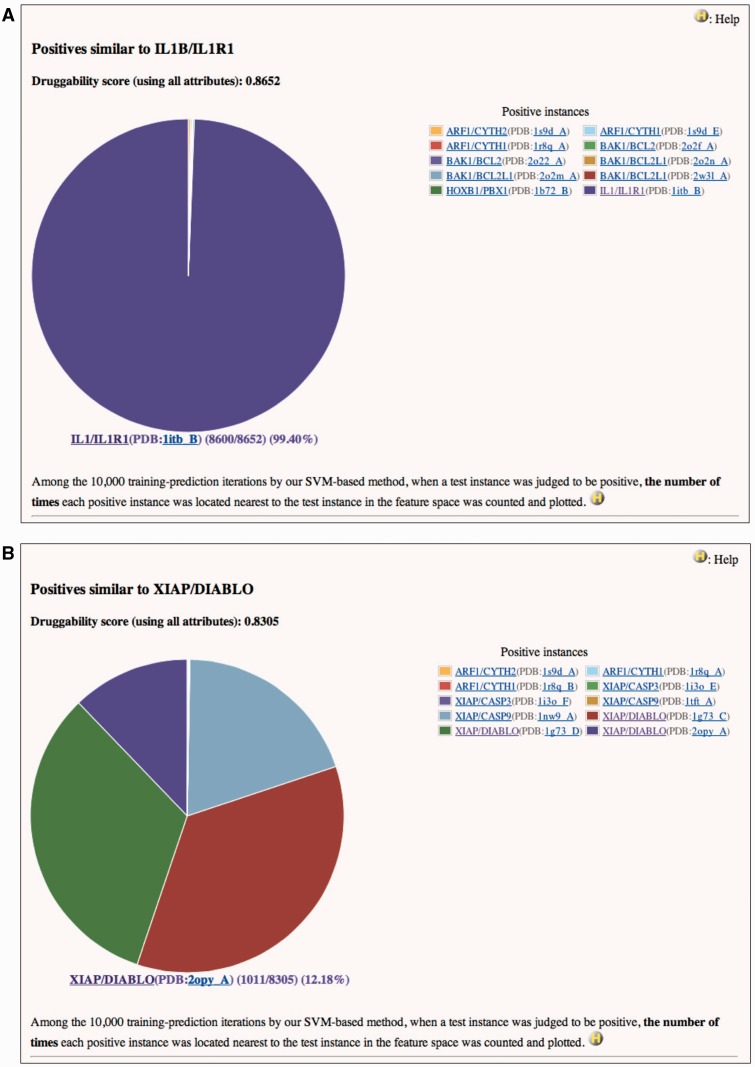
Pie charts of the number of times each positive instance (see ‘Legend’ of the chart) was located nearest to (**A**) IL1B/IL1R1 or (**B**) XIAP/DIABLO in a feature space, when IL1B/IL1R1 or XIAP/DIABLO was assessed by our SVM-based method. (A) Druggability score (using all attributes) of IL1B/IL1R1 is 0.8652. This means that IL1B/IL1R1 was judged to be positive 8652 times in the 10 000 training-prediction iteration. Among the 8652, IL1B/IL1R1 is 8600 times most closely located to itself in the feature space. Structural attributes are based on the PDB entry 1ITB. This is a screenshot of http://www.drpias.net/view_similar_positives.php?attr=all_attr&interaction_id=28988. (B) Druggability score (using all attributes) of XIAP/DIABLO is 0.8305. This means that XIAP/DIABLO was judged to be positive 8305 times in the 10 000 training-prediction iteration. Among the 8305, 4 positive instances (XIAP/CASP9(PDB:1nw9_A), XIAP/DIABLO(PDB:1g73_C), XIAP/DIABLO(PDB:1g73_D), and XIAP/DIABLO(PDB:2opy_A)) are 1011–2729 times most closely located to XIAP/DIABLO in the feature space. Structural attributes are based on the PDB entry 1G73. This is a screenshot of http://www.drpias.net/view_similar_positives.php?attr=all_attr&interaction_id=3100.

Two examples are shown in Figure 1. Both IL1B/IL1R1 and XIAP/DIABLO are the positives and also are used as the tests in this case. They have high druggability scores of 0.8652 and 0.8305, respectively, when assessed using all attributes. It is reasonable that the feature vector of IL1B/IL1R1 is essentially identical to itself (Figure 1A). On the other hand, Figure 1B indicates that the feature vectors of XIAP/DIABLO have high frequencies to be located nearest to each other in the feature space of SVM. Similarity matrix of the positives based on all attributes (Supplementary Table S3) clearly shows that the identical PPIs (only the structural attributes are different) and the homologous PPIs form a cluster in the feature space. This trend is also observed in similarity matrices based on structural (Supplementary Table S4), drug/chemical (Supplementary Table S5) and functional attributes (Supplementary Table S6). [In these tables, similarity scores range from 0 (dissimilar) to 10 000 (highly similar or identical).] In these matrices, however, similarities not only within clusters but also between clusters are observed. For example, in Supplementary Table S4, all instances in the cluster of EGFR/GRB2 and GRB2/MET show slight similarities (scores of 258–825) to STAT3/STAT3 (see No. 43–60 in row and No. 92 in column). This implies that the physicochemical properties of the pockets at the interfaces of these PPIs are similar to each other. Indeed, SH2 is common to these PPIs as the target domain for the inhibitors. Any inhibitor of EGFR/GRB2 and GRB2/MET may thus provide a starting point for the discovery or development of a small compound that can interfere with STAT3/STAT3, and vice versa. The information on similar positives may also be useful for avoiding small compounds that have a potency to inhibit non-target PPIs as well as an intended target and cause side effects. In Supplementary Table S4, instances in some clusters, such as IL1B/IL1R1 (No. 62) (also shown in Figure 1A), RAC1/TIAM1 (No. 81–84) and RAC1/TRIO (No. 85), show similarities only to themselves or only within clusters. Small compound inhibitors of these PPIs may have low potencies to bind other PPI interfaces and lead to side effects.

## Discussion

To date, researchers can access a huge amount of information on a few hundreds of drug target proteins, such as membrane receptors and enzymes. These data have been accumulated in public databases and literatures over a period of decades. In contrast, there is little information on drug target PPIs, despite the tens of thousands of experimentally identified human PPIs. Dr. PIAS will help researchers effectively explore potentially druggable PPIs by mining interactome data, thereby leading to the discovery of promising compounds that inhibit PPIs.

Some databases and webservers, for example, ANCHOR (20) and sc-PDB (21), focusing on tertiary structure of druggable ligand-binding sites and PPI interfaces will be helpful for researchers to develop or discover small compounds, by *in silico* methods, that inhibit PPIs predicted as druggable in Dr. PIAS. Although Dr. PIAS can assess whether a PPI is druggable or not, it does not provide users with tools for *in silico* drug design and is not suitable for more detailed dissection of tertiary structure of PPI interfaces. Users can search for druggable PPIs in Dr. PIAS as the first step and then can use other databases and webservers described above to design a drug targeting the PPIs as the second step. The cooperative use of Dr. PIAS and other resources will facilitate the discovery of drugs targeting PPIs.

## Supplementary data

Supplementary data are available at *Database* Online.

## Funding

Funding for open access charge: PharmaDesign, Inc.
